# Short-Term Outcomes of Patients with Non-Metastatic Malignant Solid Tumor after Coronary Artery Bypass Grafting: A Population-Based Study of National/Nationwide Inpatient Sample From 2015 To 2020

**DOI:** 10.21470/1678-9741-2024-0202

**Published:** 2025-05-23

**Authors:** Renxi Li, Deyanira J. Prastein

**Affiliations:** 1 The George Washington University School of Medicine and Health Sciences, Washington, D.C., United States of America; 2 Department of Surgery, The George Washington University Hospital, Washington, D.C., United States of America

**Keywords:** Coronary Artery Bypass, Thoracic Surgery, Neoplasms, Risk, Mortality, Morbidity, Length of Stay

## Abstract

**Introduction:**

Previous studies found that patients with a history of cancer either have similar outcomes or face an increased risk of early morbidity following cardiac surgery. However, the applicability of these findings to clinical practice may be constrained by the heterogeneity of cancer patients. To refine our understanding, this study focuses specifically on the in-hospital outcomes of patients with non-metastatic malignant solid tumors (NMST) undergoing coronary artery bypass grafting (CABG).

**Methods:**

Patients who underwent CABG were identified in National/Nationwide Inpatient Sample from Q4 2015-2020. Exclusion criteria included age < 18 years, concomitant procedures, and other malignancies. A 1:3 propensity-score matching was employed to address differences in demographics, socioeconomic status, primary payer status, hospital characteristics, comorbidities, and admission status between patients with and without NMST. In-hospital outcomes after CABG were evaluated.

**Results:**

There were 2,139 patients with NMST who underwent CABG and who were matched to 6,580 out of 164,351 patients without NMST. Patients with and without NMST had comparable mortality (2.25% vs. 2.16%, P=0.80). However, NMST patients have a higher risk of hemorrhage/hematoma (63.48% vs. 58.27%, P<0.01) and a higher rate of transfer out (28.75% vs. 25.36%, P<0.01). In addition, patients with NMST had longer time from admission to operation (P<0.01), a longer length of stay (P<0.01), and higher hospital charges (P<0.01).

**Conclusion:**

Patients with NMST have comparable short-term outcomes after CABG, except for a higher risk of postoperative bleeding. Thus, CABG could be performed safely for NMST patients, despite long-term prognosis of these patients may require further investigation.

## INTRODUCTION

**Table:** 

Abbreviations, Acronyms & Symbols				
AKI	= Acute kidney injury		MACE	= Major adverse cardiovascular event
CABG	= Coronary artery bypass grafting		MI	= Myocardial infarction
CAD	= Coronary artery disease		NIS	= National/Nationwide Inpatient Sample
CVA	= Cerebrovascular accident		NMST	= Non-metastatic malignant solid tumors
ICD-10-CM	= International Classification of Diseases, 10^th^ Revision, Clinical Modification		PCI PE	= Percutaneous coronary intervention = Pulmonary embolism
ICD-10-PCS	= International Classification of Diseases, 10^th^ Revision, Procedure Coding System		SD TIA	= Standard deviation = Transient ischemic attack
LOS	= Length of stay		VTE	= Venous thromboembolism

Coronary artery disease (CAD), affecting an estimated 16.5 million Americans, stands as a primary cause of mortality in the United States of America^[1]^. As a prevalent coronary revascularization treatment of CAD, coronary artery bypass grafting (CABG) is performed in over 200,000 patients annually in the country^[2]^.

Over the past two decades, there has been a significant increase in both the number and life expectancy of cancer survivors^[3^,^4]^. This trend has led to a corresponding rise in the incidence of comorbid cancer among patients undergoing CABG^[5]^. The decision-making process for determining CABG candidacy can be intricate in patients with a history of cancer. These individuals often have a higher risk profile due to systemic illness, potential reductions in life expectancy, and the impacts of cancer therapies on their overall health. Furthermore, the physiological stress induced by major surgeries like CABG can influence oncological outcomes by modifying the immune response^[6]^.

Previous research suggests that patients with a history of cancer either have outcomes similar to those without cancer or face an increased risk of early morbidity but not mortality following cardiac surgery^[5^,^7^,^8]^. However, the applicability of these findings to clinical practice may be constrained by the heterogeneity of cancer patients in these studies. To refine our understanding, this study focuses specifically on the in-hospital outcomes of patients with non-metastatic malignant solid tumors (NMST) undergoing CABG. The National/Nationwide Inpatient Sample (NIS) dataset, the largest national inpatient registry in the United States of America, was used to provide a robust, population-based analysis.

## METHODS

### Data Source

The NIS database from the last quarter of 2015 to 2020 was used. Patients who underwent CABG were identified using the International Classification of Diseases, 10^th^ Revision, Procedure Coding System (ICD-10-PCS) codes of 0210xxx. Patients with NMST were further selected by International Classification of Diseases, 10^th^ Revision, Clinical Modification (ICD-10-CM) codes of C00-C14, C15-C26, C30-C41, C43-C49, C4A, C50-C58, C60-C76, C7A, D46.9, E31.21, E31.22, and E31.23 by the Elixhauser Comorbidity^[9]^.

Exclusion criteria included those who have age < 18 years, concomitant procedures including aortic valve replacement (ICD-10-PCS 02RFxxx) and mitral valve replacement (ICD-10-PCS 02RGxxx), and other malignancy including lymphoma (ICD-10-CM C81-C86, C88, C90.0, C90.2, C90.3, C96.0, C96.2, C96.4, C96.9, C96.A, C96.Z, D47.Z9), leukemia (ICD-10-CM C90.1, C91-C95), solid tumor without metastasis in situ (ICD-10-CM D00-D07, D09), and metastatic cancer (ICD-10-CM C77-C79, C7B, C80.0)^[9]^. Patients with and without NMST were stratified into two cohorts in this study.

### Preoperative Factors

Preoperative factors in patients with and without NMST are shown in Table 1 and Table 2. Table 1 lists demographics (sex, age, and race and ethnicity), socioeconomic status, primary payer status, hospital characteristics, transfer status, and admission status. The average household income from the patient's ZIP code was estimated. Patients were then stratified into four quartiles based on the income of their neighborhood. Hospital characteristics included hospital bed size, location, and teaching status. American Hospital Association's yearly survey, hospital location, and teaching status of the hospital were used to stratify the hospital bed sizes into small, medium, and large. Table 2 includes the patient’s comorbidities and relevant diagnoses. Comorbidities were identified by Elixhauser measure and by ICD-10-CM codes as listed in Table S1^[9]^.

**Table 1:** Comparing demographics, primary payer status, hospital characteristics, and admission type between patients with and without NMST who underwent CABG before and after 1:3 propensity-score matching.

	NMST (n = 2,139)	Pre-match		Post-match	
		Control (n = 164,351)	*P*-value	Control (n = 6,580)	*P*-value
Sex					
Male	1713 (80.08%)	124288 (75.62%)	< 0.01	5340 (81.16%)	0.28
Female	426 (19.92%)	40049 (24.37%)	< 0.01	1240 (18.84%)	0.28
Age					
< 55 years	79 (3.69%)	22885 (13.92%)	< 0.01	266 (4.04%)	0.52
≤ 55 to < 65 years	402 (18.79%)	48156 (29.3%)	< 0.01	1248 (18.97%)	0.90
≤ 65 to < 75 years	935 (43.71%)	61135 (37.2%)	< 0.01	2889 (43.91%)	0.90
≤75 to < 85 years	654 (30.58%)	29584 (18%)	< 0.01	1975 (30.02%)	0.63
≥ 85 years	69 (3.23%)	2591 (1.58%)	< 0.01	202 (3.07%)	0.77
Race and Ethnicity					
Caucasian	1688 (78.92%)	122899 (74.78%)	< 0.01	5194 (78.94%)	0.98
African American	153 (7.15%)	11447 (6.96%)	0.73	490 (7.45%)	0.70
Hispanic	113 (5.28%)	12633 (7.69%)	< 0.01	342 (5.2%)	0.87
Asian	44 (2.06%)	5410 (3.29%)	< 0.01	143 (2.17%)	0.80
Native Americans	9 (0.42%)	889 (0.54%)	0.55	28 (0.43%)	1.00
Other races	60 (2.81%)	4792 (2.92%)	0.85	161 (2.45%)	0.34
Socioeconomic Status					
Income 1^st^ quartile (0-25%)	524 (24.5%)	45312 (27.57%)	< 0.01	1575 (23.94%)	0.64
Income 2^n^d quartile (25-50%)	580 (27.12%)	44477 (27.06%)	0.96	1824 (27.72%)	0.62
Income 3^rd^ quartile (50-75%)	530 (24.78%)	39549 (24.06%)	0.45	1610 (24.47%)	0.79
Income 4^th^ quartile (75-100%)	466 (21.79%)	32192 (19.59%)	0.01	1462 (22.22%)	0.72
Primary Payer Status					
Medicare	1514 (70.78%)	88965 (54.13%)	< 0.01	4562 (69.33%)	0.21
Medicaid	90 (4.21%)	12990 (7.9%)	< 0.01	287 (4.36%)	0.81
Private insurance	441 (20.62%)	51576 (31.38%)	< 0.01	1446 (21.98%)	0.18
Self-pay	16 (0.75%)	4828 (2.94%)	< 0.01	54 (0.82%)	0.89
No charge	6 (0.28%)	516 (0.31%)	1.00	17 (0.26%)	0.81
Other payment	70 (3.27%)	5231 (3.18%)	0.80	206 (3.13%)	0.72
Hospital characteristics					
Rural hospital	37 (1.73%)	4508 (2.74%)	< 0.01	114 (1.73%)	1.00
Urban private practice	294 (13.74%)	25880 (15.75%)	0.01	940 (14.29%)	0.50
Urban teaching hospital	1808 (84.53%)	133963 (81.51%)	< 0.01	5526 (83.98%)	0.52
Small bed size	229 (10.71%)	18811 (11.45%)	0.30	679 (10.32%)	0.60
Medium bed size	561 (26.23%)	43795 (26.65%)	0.68	1673 (25.43%)	0.48
Large bed size	1349 (63.07%)	101745 (61.91%)	0.28	4228 (64.26%)	0.32
Admission type					
Elective	1079 (50.51%)	75595 (46.15%)	< 0.01	3346 (50.85%)	0.80
Urgent	1057 (49.42%)	88219 (53.68%)	< 0.01	3234 (49.15%)	0.80

CABG=coronary artery bypass grafting; NMST=non-metastatic malignant solid tumor

**Table 2:** Comparing comorbidities and relevant diagnoses between patients with and without NMST who underwent CABG before and after 1:3 propensity-score matching.

	NMST (n = 2,139)	Pre-match		Post-match	
		Control (n = 164,351)	*P*-value	Control (n = 6,580)	*P*-value
Comorbidities and relevant diagnoses					
Acquired immune deficiency syndrome	10 (0.47%)	599 (0.36%)	0.37	22 (0.33%)	0.41
Alcohol abuse	88 (4.11%)	5580 (3.4%)	0.07	276 (4.19%)	0.95
Autoimmune conditions	43 (2.01%)	4126 (2.51%)	0.16	118 (1.79%)	0.52
Cerebrovascular disease	44 (2.06%)	3261 (1.98%)	0.81	121 (1.84%)	0.52
Heart failure	0 (0%)	140 (0.09%)	0.43	0 (0%)	NA
Dementia	30 (1.4%)	1726 (1.05%)	0.11	86 (1.31%)	0.74
Depression	200 (9.35%)	14508 (8.83%)	0.40	603 (9.16%)	0.83
Diabetes without chronic complications	335 (15.66%)	27721 (16.87%)	0.15	1058 (16.08%)	0.68
Diabetes with chronic complications	631 (29.5%)	53060 (32.28%)	0.01	1864 (28.33%)	0.32
Drug abuse	31 (1.45%)	2904 (1.77%)	0.32	106 (1.61%)	0.69
Complicated hypertension	878 (41.05%)	58708 (35.72%)	< 0.01	2660 (40.43%)	0.61
Uncomplicated hypertension	996 (46.56%)	85691 (52.14%)	< 0.01	3083 (46.85%)	0.80
Moderate to advanced liver disease	7 (0.33%)	136 (0.08%)	< 0.01	19 (0.29%)	0.82
Chronic pulmonary disease	554 (25.9%)	35513 (21.61%)	< 0.01	1663 (25.27%)	0.57
Obesity	482 (22.53%)	48018 (29.22%)	< 0.01	1464 (22.25%)	0.77
Paralysis	33 (1.54%)	2336 (1.42%)	0.65	87 (1.32%)	0.45
Peripheral vascular disease	418 (19.54%)	23004 (14%)	< 0.01	1265 (19.22%)	0.80
Advanced renal failure	41 (1.92%)	3948 (2.4%)	0.15	129 (1.96%)	1.00
Hypothyroidism	267 (12.48%)	18012 (10.96%)	0.03	802 (12.19%)	0.70
Other thyroid disorders	22 (1.03%)	1671 (1.02%)	0.91	59 (0.9%)	0.60
Valvular diseases	13 (0.61%)	856 (0.52%)	0.54	42 (0.64%)	1.00
Left ventricular dysfunction	8 (0.37%)	460 (0.28%)	0.40	30 (0.46%)	0.71
Pulmonary hypertension	100 (4.68%)	7352 (4.47%)	0.64	306 (4.65%)	0.95
Endocarditis	6 (0.28%)	182 (0.11%)	0.04	19 (0.29%)	1.00
Nonrheumatic mitral valve disorders	169 (7.9%)	12682 (7.72%)	0.74	501 (7.61%)	0.64
Nonrheumatic aortic valve disorders	110 (5.14%)	6497 (3.95%)	0.01	330 (5.02%)	0.82
Nonrheumatic tricuspid valve disorders	12 (0.56%)	1213 (0.74%)	0.44	42 (0.64%)	0.87
Nonrheumatic pulmonary valve disorders	5 (0.23%)	262 (0.16%)	0.40	12 (0.18%)	0.58
Atrial fibrillation	852 (39.83%)	55011 (33.47%)	< 0.01	2643 (40.17%)	0.78
Atrial flutter	134 (6.26%)	6985 (4.25%)	< 0.01	382 (5.81%)	0.43
Ventricular fibrillation	45 (2.1%)	3356 (2.04%)	0.82	144 (2.19%)	0.86
Ventricular flutter	1 (0.05%)	27 (0.02%)	0.30	4 (0.06%)	1.00
Sick sinus syndrome	36 (1.68%)	2230 (1.36%)	0.19	116 (1.76%)	0.85
First-degree atrioventricular block	37 (1.73%)	2342 (1.42%)	0.23	112 (1.7%)	0.92
Second-degree atrioventricular block	15 (0.7%)	977 (0.59%)	0.48	47 (0.71%)	1.00
Complete atrioventricular block	30 (1.4%)	1984 (1.21%)	0.42	105 (1.6%)	0.61
Carotid artery disease	218 (10.19%)	12079 (7.35%)	< 0.01	631 (9.63%)	0.45
Hyperlipidemia	1662 (77.7%)	133099 (80.98%)	< 0.01	5155 (78.34%)	0.51
Anemia	138 (6.45%)	7245 (4.41%)	< 0.01	425 (6.46%)	1.00
Thrombocytopenia	452 (21.13%)	31245 (19.01%)	0.01	1384 (21.03%)	0.98
Sleep apnea	308 (14.4%)	25732 (15.66%)	0.12	936 (14.22%)	0.83
Tobacco use	1132 (52.92%)	83369 (50.73%)	0.05	3451 (52.45%)	0.75
Previous MI	359 (16.78%)	29957 (18.23%)	0.09	1082 (16.44%)	0.74
Previous CVA	331 (15.47%)	28535 (17.36%)	0.02	1000 (15.2%)	0.78
Previous CABG	43 (2.01%)	3582 (2.18%)	0.65	131 (1.99%)	0.93
Previous PCI	162 (7.57%)	11633 (7.08%)	0.37	463 (7.04%)	0.44
Previous valve replacement	8 (0.37%)	501 (0.3%)	0.55	26 (0.4%)	1.00

CABG=coronary artery bypass grafting; CVA=cerebrovascular accident; MI=myocardial infarction; NMST=non-metastatic malignant solid tumor; PCI=percutaneous coronary intervention

**Table S1:** The International Classification of Diseases, 10^th^ Revision, Clinical Modification (ICD-10-CM) and International Classification of Diseases, 10^th^ Revision, Procedure Coding System (ICD-10-PCS) codes for comorbidities and relevant diagnosis.

Comorbidities and Diagnosis	ICD-10-CM and ICD-10-PCS
Acquired immune deficiency syndrome	Elixhauser comorbidity
Alcohol abuse	Elixhauser comorbidity
Autoimmune conditions	Elixhauser comorbidity
Lymphoma	Elixhauser comorbidity
Leukemia	Elixhauser comorbidity
Metastatic cancer	Elixhauser comorbidity
Solid tumor without metastasis, in situ	Elixhauser comorbidity
Solid tumor without metastasis, malignant	Elixhauser comorbidity
Cerebrovascular disease	Elixhauser comorbidity
Heart failure	Elixhauser comorbidity
Dementia	Elixhauser comorbidity
Depression	Elixhauser comorbidity
Diabetes without chronic complications	Elixhauser comorbidity
Diabetes with chronic complications	Elixhauser comorbidity
Drug abuse	Elixhauser comorbidity
Complicated hypertension	Elixhauser comorbidity
Uncomplicated hypertension	Elixhauser comorbidity
Moderate to advanced liver disease	Elixhauser comorbidity
Chronic pulmonary disease	Elixhauser comorbidity
Obesity	Elixhauser comorbidity
Paralysis	Elixhauser comorbidity
Peripheral vascular disease	Elixhauser comorbidity
Advanced renal failure	Elixhauser comorbidity
Hypothyroidism	Elixhauser comorbidity
Other thyroid disorders	Elixhauser comorbidity
Valvular diseases	Elixhauser comorbidity
Left ventricular dysfunction	I50.1
Pulmonary hypertension	I27.2
Endocarditis	I33, I38, I39
Nonrheumatic mitral valve disorders	I34
Nonrheumatic aortic valve disorders	I35
Nonrheumatic tricuspid valve disorders	I36
Nonrheumatic pulmonary valve disorders	I37
Atrial fibrillation	I48.0, I48.1, I48.2, I48.91
Atrial flutter	I48.3, I48.4, I48.92
Ventricular fibrillation	I49.01
Ventricular flutter	I49.02
Sick sinus syndrome	I49.5
First-degree atrioventricular block	I44.0
Second-degree atrioventricular block	I44.1
Complete atrioventricular block	I44.2
Carotid artery disease	I65.21, I65.22, I65.23, I65.29
Hyperlipidemia	E78.0, E78.1, E78.2, E78.3, E78.5, E78.5
Anemia	D63
Thrombocytopenia	D69.5, D69.6
Sleep apnea	G47.3
Tobacco use	F17, Z72.0, Z87.891
Previous myocardial infarction	I25.2
Previous cerebrovascular accident	Z86.73
Previous coronary artery bypass grafting	Z95.1
Previous percutaneous coronary intervention	Z98.61, Z95.5
Previous valve replacement	Z95.2, Z95.3, Z95.4

### Postoperative Outcomes

In-hospital outcomes after CABG were examined (Table 3). The outcomes included mortality, major adverse cardiovascular event (MACE), myocardial infarction (MI), stroke, transient ischemic attack (TIA), neurological complications, pericardial complications, pacemaker implantation, cardiogenic shock, respiratory complications, mechanical ventilation, acute kidney injury (AKI), postprocedural renal failure, venous thromboembolism (VTE), pulmonary embolism (PE), hemorrhage/hematoma, infection, sepsis, deep wound complication, superficial wound complication, vascular complication, diaphragmatic paralysis, and reopen surgery for bleeding control. Moreover, transfer out to other facilities, time from admission to operation, hospital length of stay (LOS), and total hospital charge were compared between patients with and without NMST. The ICD-10-CM/PCS codes used to define the outcomes are shown in Table S2.

**Table S2:** The International Classification of Diseases, 10^th^ Revision, Clinical Modification (ICD-10-CM) and International Classification of Diseases, 10^th^ Revision, Procedure Coding System (ICD-10-PCS) codes for perioperative outcomes.

Perioperative outcomes	ICD-10-CM and ICD-10-PCS
MACE	
MI	I97.710, I97.711, I97.120, I97.121, I46.9
Stroke	I60.9, I61.9, I63.22, I63.139, I63.239, I63.019, I63.119, I63.219, I97.811, I97.820, I97.821, I97.810
Postprocedural cardiogenic shock	T81.11XA
Postprocedural heart failure	I97.130, I97.131
Postprocedural cardiac insufficiency	I97.110, I97.111
MI	I97.710, I97.711, I97.120, I97.121, I46.9
Stroke	I60.9, I61.9, I63.22, I63.139, I63.239, I63.019, I63.119, I63.219, I97.811, I97.820, I97.821, I97.810
Transient ischemic attack	G45.9, I67.848
Neurological complications	
Nervous system complication, unspecified	G97.81
Central nervous system complication	G97.81, G97.82
Iatrogenic cerebrovascular infarction or hemorrhage	I97.811, I97.821, I97.810, I97.820
Transient ischemic attack	G45.9, I67.848
Any stroke	160.9, 161.9, 163.22, 163.139, 163.239; 163.019, 163.119, 163.219
Pericardial complications	
Hemopericardium	I31.2
Tamponade	I31.4
Pericardiocentesis	0W9DXXX, 0W9CXXX, 0W9D40Z
Acute pericarditis	I30.1, I30.8, I30.9
Pacemaker implantation	0JH606Z, 0JH636Z, 0JH806Z, 0JH836Z, 0JH60PZ, 0JH63PZ, 0JH80PZ, 0JH83PZ, 0JH604Z, 0JH634Z, 0JH804Z, 0JH834Z, 0JH605Z, 0JH635Z, 0JH805Z, 0JH835Z, 02H73KZ, 02HK3KZ, 02HL3KZ, 02HN0KZ, 02HN4KZ, 0JH608Z, 0JH638Z, 0JH808Z, 0JH838Z, 02H60KZ, 02H63KZ, 02H64KZ, 02H70KZ, 02H73KZ, 02H74KZ, 02HK0KZ, 02HK3KZ, 02HK4KZ, 02HL0KZ, 02HL3KZ, 02HL4KZ, 0JH608Z, 0JH638Z, 0JH808Z, 0JH838Z, 02H60KZ, 02H63KZ, 02H64KZ, 02H70KZ, 02H73KZ, 02H74KZ, 02HK0KZ, 02HK3KZ, 02HK4KZ, 02HL0KZ, 02HL3KZ, 02HL4KZ, 0JH608Z, 0JH638Z, 0JH808Z, 0JH838Z,
Cardiogenic shock	R57.0
Respiratory complications	
Pneumothorax/hemothorax	J95.811, J95.812, J95.830, J95.831, J94.2
Diaphragm paralysis	J98.6
Postoperative respiratory failure	J95.821, J96.00, J95.822, J96.20
Pulmonary insufficiency	J95.2, J95.3
Respiratory arrest	R09.2
Other iatrogenic respiratory complications	J95.88, J95.89, J95.850, J95.851, J95.859
Mechanical ventilation	5A1935Z, 5A1945Z, 5A1955Z
Acute kidney injury	N17.9, N17.0, N17.1, N17.2
Postprocedural renal failure	N99.0
Venous thromboembolism	I82.401, I82.402, I82.403, I82.409, I82.411, I82.412, I82.413, I82.419, I82.421, I82.422, I82.423, I82.429, I82.431, I82.432, I82.433, I82.439, I82.441, I82.442, I82.443, I82.449, I82.451, I82.452, I82.453, I82.459, I82.461, I82.462, I82.463, I82.469, I82.491, I82.492, I82.493, I82.499, I82.4Y1, I82.4Y2, I82.4Y3, I82.4Y9, I82.4Z1, I82.4Z2, I82.4Z3, I82.4Z9
Pulmonary embolism	I26.02, I26.09, I26.92, I26.93, I26.94
Hemorrhage/hematoma	
Hemorrhage/hematoma complicating a procedure	I97.411, I97.418, I97.42, I97.611, I97.618, I97.620, I97.411, I97.418, I97.42, I97.621, I97.631, I97.638
Acute post-hemorrhagic anemia	D62
Hemorrhage requiring transfusion	3023XXX, 3024XXX
Infection	
Fever	T82.6, T82.7, R50.82
Septicemia	A41.9, A65.20, T81.12XA
Postprocedural aspiration pneumonia	J95.89
Sepsis	T81.12XA, T81.44XA
Deep wound complication	T81.32XA, T81.43XA
Superficial wound complications	L76.34, L76.32, T81.31XA, T81.41XA, T81.42XA, T81.40XA
Vascular complication	
Accidental puncture or laceration during a procedure	I97.51, I97.52
Injury to blood vessels	S25.X, S35.X
Arteriovenous fistula	I77.0
Injury to retroperitoneum	S36.899A
Vascular complication requiring surgical/percutaneous repair	03QY0ZZ, 03QY3ZZ, 03QY4ZZ, 04QY0ZZ, 04QY3ZZ, 04QY4ZZ, 05QY0ZZ, 05QY3ZZ, 05QY4ZZ, 06QY0ZZ, 06QY3ZZ, 06QY4ZZ, 02QW0ZZ, 02QW3ZZ, 02QX4ZZ, 03Q00ZZ, 03Q03ZZ, 03Q04ZZ, 03Q10ZZ, 03Q13ZZ, 03Q14ZZ, 03Q20ZZ, 03Q23ZZ, 03Q24ZZ, 03Q30ZZ, 03Q40ZZ, 03Q33ZZ, 03Q43ZZ, 03Q44ZZ, 03Q50ZZ, 03Q53ZZ, 03Q54ZZ, 03Q60ZZ, 03Q63ZZ, 03Q64ZZ, 03Q74ZZ 03Q70ZZ, 03Q73ZZ, 03Q80ZZ, 03Q83ZZ, 03Q84ZZ, 03Q90ZZ, 03Q93ZZ, 03Q94ZZ, 03QA0ZZ, 03QA3ZZ, 03QA4ZZ, 03QB0ZZ, 03QB3ZZ, 03QB4ZZ,03QC0ZZ, 03QC3ZZ, 03QC4ZZ,03QY0ZZ, 03QY3ZZ,03QY4ZZ,04Q00ZZ, 04Q03ZZ, 04QC0ZZ, 04QC3ZZ, 04Q04ZZ, 04QC4ZZ,04QD0ZZ, 04QD3ZZ,04QD4ZZ, 04QE0ZZ 04QE3ZZ, 04QE4ZZ, 04QF0ZZ, 04QF3ZZ, 04QF4ZZ, 04QH0ZZ, 04QH3ZZ, 04QH4ZZ,04QJ0ZZ, 04QJ3ZZ, 04QJ4ZZ,04QK0ZZ, 04QK3ZZ, 04QL0ZZ, 04QL3ZZ, 04QL4ZZ, 04QY0ZZ, 04QY3ZZ
Hemorrhage from vascular procedures	T82.838, T82.837
Other artery and vein complications	T81.72XA, T81.719A
Diaphragmatic paralysis	J98.6
Reopen surgery	0W390ZZ, 0W3B0ZZ, 0W3C0ZZ, 0W3D0ZZ, 0W3Q0ZZ

MACE=major adverse cardiovascular event; MI=myocardial infarction

**Table 3:** Comparing in-hospital outcomes between patients with and without NMST who underwent CABG after 1:3 propensity-score matching.

	NMST (n = 2,139)	Control (n = 6,580)	*P*-value
Mortality	48 (2.25%)	142 (2.16%)	0.80
MACE	65 (3.04%)	198 (3.01%)	0.94
MI	36 (1.69%)	115 (1.75%)	0.92
Stroke	17 (0.8%)	45 (0.68%)	0.56
TIA	9 (0.42%)	18 (0.27%)	0.27
Neurological complications	26 (1.22%)	63 (0.96%)	0.32
Pericardial complications	40 (1.87%)	112 (1.7%)	0.63
Pacemaker implantation	28 (1.31%)	128 (1.95%)	0.06
Cardiogenic shock	169 (7.91%)	458 (6.96%)	0.15
Respiratory complications	222 (10.39%)	697 (10.59%)	0.81
Mechanical ventilation	203 (9.5%)	596 (9.06%)	0.55
AKI	526 (24.63%)	1609 (24.45%)	0.88
Postprocedural renal failure	15 (0.7%)	36 (0.55%)	0.42
VTE	18 (0.84%)	50 (0.76%)	0.67
PE	2 (0.09%)	3 (0.05%)	0.60
Hemorrhage/hematoma	1356 (63.48%)	3834 (58.27%)	< 0.01
Infection	94 (4.4%)	232 (3.53%)	0.07
Sepsis	0 (0%)	4 (0.06%)	0.58
Deep wound complication	3 (0.14%)	16 (0.24%)	0.59
Superficial wound complication	27 (1.26%)	58 (0.88%)	0.13
Vascular complication	13 (0.61%)	44 (0.67%)	0.88
Diaphragmatic paralysis	4 (0.19%)	16 (0.24%)	0.80
Reopen surgery	18 (0.84%)	57 (0.87%)	1.00
Transfer out	614 (28.75%)	1669 (25.36%)	< 0.01
	Mean ± SD	Mean ± SD	*P*-value
Admission to operation (days)	2.74 ± 3.97	2.30 ± 3.06	< 0.01
LOS (days)	10.62 ± 7.55	9.87 ± 6.96	< 0.01
Total hospital charge (US$)	245,370 ± 198,736	224,851 ± 195,042	< 0.01

AKI=acute kidney injury; CABG=coronary artery bypass grafting; LOS=length of stay; MACE=major adverse cardiovascular event; MI=myocardial infarction; NMST=non-metastatic malignant solid tumor; PE=pulmonary embolism; SD=standard deviation; TIA=transient ischemic attack; VTE=venous thromboembolism

### Ethics Approval

This study was exempt from the IRB approval by The George Washington University as it analyzed retrospective, deidentified NIS data.

### Statistical Analysis

Fisher's exact test was used to compare the preoperative factors between patients with and without NMST. To account for differences in the preoperative factors between the NMST and non-NMST patients as well as a significant difference in their sample sizes, a 1:3 ratio (NMST: non-NMST) propensity-score matching was carried out using the Greedy Matching algorithm with a 2% caliper. After the propensity-score matching, Fisher's exact test was used to examine binary postoperative outcomes while two-tailed independent *t*-tests were used to compare continuous variables.

All statistical analyses were conducted using SAS, version 9.4. A *P*-value < 0.05 was considered statistically significant. The authors had full access to the NIS dataset and took responsibility for the integrity of all analyses. This is a retrospective study that used a de-identified NIS dataset. As a result, it was exempted from The George Washington University Institutional Review Board (or IRB) review.

## RESULTS

There were 2,139 patients with NMST who underwent CABG from the last quarter of 2015 to 2020. During the same time, there were 164,351 patients without NMST who underwent CABG, and 6,580 of them were matched to those with NMST.

Table 1 summarizes demographics, primary payer status, hospital characteristics, and admission type between patients with and without NMST who underwent CABG. Compared to those without NMST, patients with NMST were more likely to be males (80.08% *vs.* 75.62%, *P*<0.01), have age > 75 years (75-85 years, 43.71% *vs.* 37.20%, *P*<0.01; > 85 years, 3.23% *vs.* 1.58%, *P*<0.01), be Caucasian (78.92% *vs.* 74.78%, *P*<0.01), have income at the highest quartile (75-100%) (21.79% *vs.* 19.59%, *P*=0.01), use Medicare (70.78% *vs.* 54.13%, *P*<0.01), stay in an urban teaching hospital (84.53% *vs.* 81.51%, *P*<0.01), and under elective admission (50.51% *vs.* 46.15%, *P*<0.01). In contrast, patients with NMST were less likely to be females (19.92% *vs.* 24.37%, *P*<0.01), have age < 65 years (55-65 years, 18.79% *vs.* 29.30%, *P*<0.01; < 55 years, 3.69% *vs.* 13.92%, *P*<0.01), be Hispanic (5.28% *vs.* 7.69%, *P*<0.01) or Asian (2.06% *vs.* 3.29%, *P*<0.01), have income at the lowest quartile (0-25%) (24.50% *vs.* 27.57%, *P*<0.01), use Medicaid (4.21% *vs.* 7.90%, *P*<0.01), private insurance (20.62% *vs.* 31.38%, *P*<0.01), or self-pay (0.75% *vs.* 2.94%, *P*<0.01) as the primary payer, stay in a rural hospital (1.73% *vs.* 2.74%, *P*<0.01) or an urban private practice (13.74% *vs.* 15.75%, *P*=0.01), or under urgent admission (49.42% *vs.* 53.68%, *P*<0.01). All preoperative differences were matched by 1:3 propensity-score matching.

Table 2 shows the comparison of comorbidities and relevant diagnoses between patients with and without NMST who underwent CABG. Patients with NMST were more likely to have complicated hypertension (41.05% *vs.* 35.72%, *P*<0.01), moderate to advanced liver disease (0.33% *vs.* 0.08%, *P*<0.01), chronic pulmonary disease (25.90% *vs.* 21.61%, *P*<0.01), peripheral vascular disease (19.54% *vs.* 14.00%, *P*<0.01), hypothyroidism (12.48% *vs.* 10.96%, *P*=0.03), endocarditis (0.28% *vs.* 0.11%, *P*=0.04), nonrheumatic aortic valve disorders (5.14% *vs.* 3.95%, *P*=0.01), atrial fibrillation (39.83% *vs.* 33.47%, *P*<0.01), atrial flutter (6.26% *vs.* 4.25%, *P*<0.01), anemia (6.45% *vs.* 4.41%, *P*<0.01), and thrombocytopenia (21.13% *vs.* 19.01%, *P*=0.01). In contrast, patients with NMST were less likely to have diabetes with chronic complications (29.50% *vs.* 32.28%, *P*=0.01), uncomplicated hypertension (46.56% *vs.* 52.14%, *P*<0.01), obesity (22.53% *vs.* 29.22%, *P*<0.01), hyperlipidemia (77.70% *vs.* 80.98%, *P*<0.01), or previous cerebrovascular accident (15.47% *vs.* 17.36%, *P*=0.02). All differences in the comorbidities and relevant diagnoses between NMST and non-NMST patients were matched by propensity-score matching.

Table 3 summarizes the in-hospital outcomes between patients with and without NMST who underwent CABG after 1:3 propensity-score matching. Patients with and without NMST had comparable mortality (2.25% *vs.* 2.16%, *P*=0.80). However, NMST patients have a higher risk of hemorrhage/hematoma (63.48% *vs.* 58.27%, *P*<0.01) and a higher rate of transfer out (28.75% *vs.* 25.36%, *P*<0.01). In addition, patients with NMST had longer time from admission to operation (2.74 ± 3.97 *vs.* 2.30 ± 3.06 days, *P*<0.01), longer LOS (10.62 ± 7.55 *vs.* 9.87 ± 6.96 days, *P*<0.01), and higher total hospital charges (US$245,370 ± 198,736 *vs.* US$224,851 ± 195,042, *P*<0.01). Other in-hospital outcomes, including the rates of MACE, MI, stroke, TIA, neurological complications, pericardial complications, pacemaker implantation, cardiogenic shock, respiratory complications, mechanical ventilation, AKI, postprocedural renal failure, VTE, PE, infection, sepsis, deep wound complication, superficial wound complication, vascular complication, diaphragmatic paralysis, and reopen surgery, were not different between patients with and without NMST.

## DISCUSSION

The study conducted a population-based analysis of the in-hospital outcomes of patients with NMST who underwent CABG. It was found that patients with NMST had comparable mortality and morbidity rates, except for a higher risk of hemorrhage/hematoma. Additionally, NMST patients exhibited a higher rate of transfer out, an extended duration from admission to operation, longer LOS, and increased total hospital charges.

A small case series study by Zhang et al.^[8]^ demonstrated that CABG is an effective treatment for CAD patients with malignancy. Previous population-based studies indicated that cancer patients undergoing CABG generally had comparable outcomes, with the exception of a higher incidence of postoperative major bleeding^[5^,^10]^. This aligns with the findings of the current study, where NMST patients were at an increased risk of hemorrhage/hematoma after CABG, while other in-hospital outcomes were similar to those of non-NMST patients.

Several factors may contribute to the heightened risk of bleeding in patients with NMST. Open surgery can prompt the release of pro-inflammatory cytokines that can disrupt fibrinogen and platelet function^[11]^. These disturbances can be associated with altered coagulative states in NMST patients^[11]^. Additionally, the introduction of new anticoagulation and antifibrinolytic drugs for certain cancer patients might play a role^[12^,^13]^. While these drugs can reduce ischemic risk, they may also increase the risk of bleeding in some cancer patients^[12^,^13]^. Furthermore, the use of specific anti-cancer therapies, such as selective estrogen receptor modulators in breast cancer and androgen deprivation therapy in prostate cancer, could lead to elevated bleeding risk^[12^,^13]^.

Despite the increased risk of bleeding, it is important to note that in-hospital mortality, cardiac complications, and other organ system complications were not elevated in NMST patients. This observation can be valuable for preoperative risk stratification, suggesting that CABG can be relatively safe for NMST patients, despite their long-term prognosis is still needed in future studies.

Additionally, in NMST patients, a significant delay was observed from admission to operation, even after adjusting for all preoperative factors. This delay could arise from the need for preoperative stabilization, considering their potential elevated risk profile and systemic illness. Moreover, the evaluation and decision-making process for CABG in NMST patients could be more complex, which could contribute to the postponement of surgical intervention. This delay, coupled with a higher rate of complications in NMST patients, may result in prolonged LOS and, consequently, increased total hospital charges.

### Limitations

This study has several limitations to acknowledge. Firstly, as an administrative database, the NIS does not have detailed data for the malignancy, such as tumor, node, and metastasis (or TNM) staging, cancer grade, or the use of chemotherapy, radiation therapy, and/or immunotherapy. Additionally, the NIS lacks information on various factors that could influence revascularization outcomes, including ejection fraction, specifics of coronary segments, stenosis diameter, lesion presence, coronary artery dominance, and small vessel disease^[14^,^15]^. This lack of data also precludes the calculations of surgical risk scores, such as the Society of Thoracic Surgeons score^[16]^ or the European System for Cardiac Operative Risk Evaluation (or EuroSCORE) score^[17]^. Furthermore, the NIS database is limited to in-hospital outcomes, where follow-up after discharge is not recorded. This restricts the analysis of this study to short-term outcomes and does not provide insights into the long-term prognosis of NMST patients after CABG.

## CONCLUSION

In summary, this study conducted a population-based analysis to compare the in-hospital outcomes of CABG between patients with and without NMST. It was found that patients with NMST had comparable mortality and morbidity rates, with the exception of a higher risk of hemorrhage/hematoma. In addition, NMST patients had a higher rate of transfer out, longer time from admission to operation, longer LOS, and increased total hospital charges. Therefore, this study can aid in preoperative risk stratification for patients with NMST; CABG could be performed relatively safely for these patients while additional attention should be given to postoperative bleeding, and the long-term prognosis of NMST patients may require further investigation.
